# Towards control of the global HIV epidemic: Addressing the middle-90 challenge in the UNAIDS 90–90–90 target

**DOI:** 10.1371/journal.pmed.1002293

**Published:** 2017-05-02

**Authors:** Collins Iwuji, Marie-Louise Newell

**Affiliations:** 1 Research Department of Infection and Population Health, University College London, London, United Kingdom; 2 Africa Health Research Institute, KwaZulu-Natal, South Africa; 3 Human Development and Health and Global Health Research Institute, Faculty of Medicine, University of Southampton, Southampton, United Kingdom

## Abstract

In a Perspective, Collins Iwuji and Marie-Louise Newell discuss early findings from Richard Hayes and colleagues' PopART study on HIV testing and treatment.

In 2016, just over 2 million people worldwide acquired HIV infection, mostly via heterosexual transmission and mostly in sub-Saharan Africa [1]. Antiretroviral treatment (ART) aims to suppress viral load to very low, or undetectable, levels, delaying HIV disease progression [2,3] and reducing the risk of onward transmission [4]. Following the 2015 WHO guidelines recommending ART for all HIV-positive people regardless of CD4 count, WHO and the Joint United Nations Programme on HIV/AIDS (UNAIDS) issued the 90–90–90 target, aiming by 2020 to have 90% of infected people knowing their HIV status, 90% of HIV-positive people initiated on ART, and 90% of people treated with ART virally suppressed [5], so as to achieve containment of the HIV epidemic.

We commend Richard Hayes and colleagues for their success in navigating the complex logistic challenges in implementing a large-scale universal testing and treatment (UTT) intervention in sub-Saharan Africa, as described in their accompanying research article in *PLOS Medicine* [6]. They report how close they were able to come to reaching the first two stages of the 90–90–90 target in four communities in Zambia after one year of implementing their PopART intervention (comprising home-based HIV testing by Community HIV care Providers [CHiPs] with support for linkage to care, adherence, and retention). Among those consenting to the intervention, 6,197 HIV-positive individuals not on ART (most of whom had never been in care) were referred to care, 42% of whom initiated ART within six months and 53% by 12 months. Extrapolating to the entire population, the estimated percentage of HIV-positive adults who knew their status increased from 52% to 78% (men) and from 56% to 87% (women); percentages of known HIV-positive people on ART increased from 54% to 74% (men) and from 53% to 73% (women). The overall estimated percentage of HIV-positive adults on ART was 61% after 1 y of intervention implementation, compared to the WHO/UNAIDS target of 81% (90% of 90%). We note that many process indicators in the study were based on self-report and, apparently, data from the CHiPs electronic capture system were not verified with clinic data. Further, in this setting, in which nearly 50% of all HIV-positive individuals were ART-naive because they were newly diagnosed, it is likely that most of the 20% of people who migrated out of the area never linked to care or commenced ART. This 20% of people, no longer resident in the PopART communities, was not included in the denominator for the post-CHiPs evaluation at 12 months on linkage to care and ART initiation, which could have resulted in an overestimate of the change observed following the CHiPs intervention.

Hayes and colleagues’ findings thus confirm the high acceptability of home-based HIV testing, and a less-than-optimal linkage to care, with initiation of ART rates suggesting that those who do link to care are willing to start ART; there is a substantial proportion of people identified as HIV positive who do not (yet) link to care, can therefore not be initiated on ART, and who may continue to transmit HIV. The Agence Nationale de Recherche sur le Sida et les hépatites virales (ANRS) 12249 Treatment as Prevention (TasP) trial, also evaluating a UTT intervention in rural South Africa, recently reported that 92% of HIV-positive individuals knew their status, 49% of those people initiated ART, and 93% achieved virological suppression on ART [7]. Higher rates of ART initiation were reported from Uganda and Kenya in the Sustainable East Africa Research in Community Health (SEARCH) trial, another UTT intervention study, with 97% of HIV-positive individuals knowing their status, 93% on ART, and 90% virologically suppressed at the end of year two [8]. Similarly, estimates from the Botswana Combination Prevention Project (BCPP) trial, a UTT intervention implemented in Botswana, reported that, overall, 70% of all HIV-positive individuals were virologically suppressed, close to the UNAIDS target of 73% (90% of 90% of 90%), with 83% of HIV-positive individuals diagnosed and 87% on ART, of whom 97% achieved virological suppression [9]. The different approaches to the estimation of percentages in the HIV care cascade in these trials hinder direct comparison of results; hence, we agree with Hayes and colleagues that approaches for estimating proportions of people at different stages in the HIV cascade need to be harmonised.

Hayes and colleagues report that it was challenging to find young men at home, again in line with experience elsewhere [10]. Slow linkage to care suggests that people will only attend facilities once they prioritise doing so, and this is especially pertinent before they are driven to do so by the development of HIV symptoms and signs. An earlier study in rural KwaZulu-Natal highlighted that linkage to care was significantly less likely in those who had never been in HIV care, students in education, and those further away from the clinic, while those who had positive experience of ART in friends or family were more likely to access the trial clinic [11]. Hayes and colleagues will address progress towards the third 90% target later in their trial, but evidence from other studies suggests that once people engage with ART care, they are likely to adhere, at least in the short term [7], and that early linkage to ART care is the main hurdle in the HIV care cascade.

The big question remains whether, in the global heterosexually-driven HIV epidemic, UTT will ultimately reduce HIV incidence to levels sufficient for containment. Findings from the ANRS 12249 TasP trial in South Africa showed little impact on HIV incidence [7], likely due to the slow linkage to care. Hayes and colleagues, although suggesting success of UTT in the first round of trial implementation, do not provide information about sustainability of either HIV test offer uptake or ART adherence, issues which may be especially important in settings with high migration movements.

Overall, these results would suggest that it is unlikely that the rather optimistic forecasts, based on statistical modelling [12], of an imminent end to the global HIV epidemic will be fulfilled. The current gloomy political environment, with uncertainty about the global will to continue to support the large-scale implementation of HIV treatment and care programmes worldwide [13], further adds to the already considerable challenges faced by public health programmes in many settings with a high HIV burden. Health care system requirements for a successful UTT programme are not negligible, even if a UTT approach is found to be cost-effective [14]. Substantial resources are needed to further scale up ART for all HIV-positive adults, and allocation of limited resources will need to be optimised on the basis of evidence of efficacy. Given extensive resource constraints, there may come a time to consider whether public programmes will need to focus on providing optimal health care and support for those people who engage with care at public facilities and who have thus indicated that they have prioritised access to health care in their lives.

## Abbreviations

ANRS: Agence Nationale de Recherche sur le Sida et les hépatites virales
ART: Antiretroviral treatment
BCPP: the Botswana Combination Prevention Project
CHiPs: Community HIV care Providers
SEARCH: Sustainable East Africa Research in Community Health
TasP: treatment as prevention
UNAIDS: the Joint United Nations Programme on HIV/AIDS
UTT: universal testing and treatment

## References

[1] UNAIDS. Global AIDS Update 2016 2016 [cited 2016 7 Jan]. http://www.unaids.org/sites/default/files/media_asset/global-AIDS-update-2016_en.pdf.

[2] Group ISS, Lundgren JD, Babiker AG, Gordin F, Emery S, Grund B, et al Initiation of Antiretroviral Therapy in Early Asymptomatic HIV Infection. N Engl J Med. 2015;373(9):795–807. doi: 10.1056/NEJMoa1506816 26192873 10.1056/NEJMoa1506816 PMC4569751

[3] Group TAS, Danel C, Moh R, Gabillard D, Badje A, Le Carrou J, et al A Trial of Early Antiretrovirals and Isoniazid Preventive Therapy in Africa. N Engl J Med. 2015;373(9):808–22. doi: 10.1056/NEJMoa1507198 26193126 10.1056/NEJMoa1507198

[4] Cohen MS, Chen YQ, McCauley M, Gamble T, Hosseinipour MC, Kumarasamy N, et al Prevention of HIV-1 infection with early antiretroviral therapy. N Engl J Med. 2011;365(6):493–505. doi: 10.1056/NEJMoa1105243 21767103 10.1056/NEJMoa1105243 PMC3200068

[5] UNAIDS. 90-90-90. An ambitious treatment target to help end the AIDS epidemic, 2014 [cited 2017, 14 March]. http://www.unaids.org/sites/default/files/media_asset/90-90-90_en_0.pdf

[6] Hayes R, Floyd S, Schaap A, Shanaube K, Bock P, Sabapathy K, et al A universal testing and treatment intervention to improve HIV control: One-year results from intervention communities in Zambia in the HPTN 071 (PopART) cluster-randomised trial. PLoS Med. 2017. 10.1371/journal.pmed.1002292 PMC5412988 28464041

[7] Iwuji C, Orne-Gliemann J, Balestre E, Larmarange J, Thiebaut R, Tanser F, et al. The impact of universal test and treat on HIV incidence in a rural South African population: ANRS 12249 TasP trial, 2012–2016. International AIDS Conference; July 18–22; Durban, South Africa 2016.

[8] Petersen M, Balzer L, Kwarsiima D, Sang N, Chamie G, Ayieko J, et al. SEARCH test and treat study in Uganda and Kenya exceeds the UNAIDS 90-90-90 cascade target by achieving 81% population-level viral suppression after 2 years. International AIDS Conference; July 18–22, 2016; Durban, South Africa.

[9] Gaolathe T, Wirth KE, Holme MP, Makhema J, Moyo S, Chakalisa U, et al Botswana's progress toward achieving the 2020 UNAIDS 90-90-90 antiretroviral therapy and virological suppression goals: a population-based survey. Lancet HIV. 2016;3(5):e221–30. doi: 10.1016/S2352-3018(16)00037-0 27126489 10.1016/S2352-3018(16)00037-0 PMC5146754

[10] Iwuji CC, Orne-Gliemann J, Larmarange J, Okesola N, Tanser F, Thiebaut R, et al Uptake of Home-Based HIV Testing, Linkage to Care, and Community Attitudes about ART in Rural KwaZulu-Natal, South Africa: Descriptive Results from the First Phase of the ANRS 12249 TasP Cluster-Randomised Trial. PLoS Med. 2016;13(8):e1002107 doi: 10.1371/journal.pmed.1002107 27504637 10.1371/journal.pmed.1002107 PMC4978506

[11] Plazy M, Farouki KE, Iwuji C, Okesola N, Orne-Gliemann J, Larmarange J, et al Access to HIV care in the context of universal test and treat: challenges within the ANRS 12249 TasP cluster-randomized trial in rural South Africa. J Int AIDS Soc. 2016;19(1):20913 doi: 10.7448/IAS.19.1.20913 27258430 10.7448/IAS.19.1.20913 PMC4891946

[12] Granich RM, Gilks CF, Dye C, De Cock KM, Williams BG. Universal voluntary HIV testing with immediate antiretroviral therapy as a strategy for elimination of HIV transmission: a mathematical model. Lancet. 2009;373(9657):48–57. doi: 10.1016/S0140-6736(08)61697-9 19038438 10.1016/S0140-6736(08)61697-9

[13] Kates J, Wexler A, Lief E. Financing the response to HIV in Low- and Middle-Income Countries: International Assistance from Donor Governments in 2014. Washington: Kaiser Family Foundation & UNAIDS 2015 [cited 2017, 14 March]. http://www.unaids.org/sites/default/files/media_asset/financing-the-response-to-HIV-in-low-and-middle-income-countries_en.pdf.

[14] Mikkelsen E, Hontelez JA, Jansen MP, Barnighausen T, Hauck K, Johansson KA, et al Evidence for scaling up HIV treatment in sub-Saharan Africa: A call for incorporating health system constraints. PLoS Med. 2017;14(2):e1002240 doi: 10.1371/journal.pmed.1002240 28222126 10.1371/journal.pmed.1002240 PMC5319640

